# Botulinum toxin injections as salvage therapy is beneficial for management of patellofemoral pain syndrome

**DOI:** 10.1186/s43019-021-00121-3

**Published:** 2021-10-29

**Authors:** Yuval Kesary, Vivek Singh, Tal Frenkel-Rutenberg, Arie Greenberg, Shmuel Dekel, Ran Schwarzkopf, Nimrod Snir

**Affiliations:** 1 Sackler Faculty of Medicine, Tel Aviv University, P. O. Box 39040, 6997801 Tel Aviv, Israel; 2 Department of Orthopedic Surgery, NYU Langone Health, NYU Langone Orthopedic Hospital, New York, NY USA; 3 Orthopedic Department, Rabin Medical Center, Beilinson Hospital, Petah Tikva, Israel; 4 Department of Orthopedic Surgery, Kaplan Medical Center, Rehovot, Israel; 5 Division of Adult Reconstruction, Department of Orthopedics, Tel Aviv Sourasky Medical Center, Tel Aviv, Israel

**Keywords:** Patella, Knee, Patellofemoral pain, Physical therapy, Botulinum toxin, Muscle imbalance

## Abstract

**Purpose:**

Patellofemoral pain syndrome (PFPS) is a common pathology usually presenting with anterior or retropatellar pain. It is associated with a relative imbalance between the vastus medialis oblique (VMO) and the vastus lateralis (VL) muscles. This can lead to considerable morbidity and reduced quality of life (QOL). This study aims to assess the long-term functional outcome of PFPS treated with VL muscle botulinum toxin A (BoNT-A) injection.

**Materials and methods:**

A retrospective review was performed on 26 consecutive patients (31 knees) with a mean age of 50.1 years (± 19.7 years) who were treated with BoNT-A injections to the VL muscle followed by physiotherapy between 2008 and 2015. Pre- and post-treatment pain levels (numerical rating scale, NRS), QOL (SF-6D), and functional scores (Kujala and Lysholm questionnaires) were measured. Demographics, physical therapy compliance, previous surgeries, perioperative complications, and patient satisfaction levels were collected.

**Results:**

The mean follow-up time was 58.8 ± 36.4 months. There were significant improvements in all the examined domains. The average pain score (NRS) decreased from 7.6 to 3.2 (*P* < 0.01), and the Kujala, Lysholm, and SF-6D scores improved from 58.9 to 82.7 (*P* < 0.001), 56.2 to 83.2 (*P* < 0.001), and 0.6 to 0.8 (*P* < 0.001), respectively. Similar delta improvement was achieved irrespective of gender, age, compliance to post-treatment physical therapy, or coexisting osteoarthritis. Patients who presented with a worse pre-treatment clinical status achieved greater improvement. Prior to BoNT-A intervention, 16 patients (18 knees) were scheduled for surgery, of whom 12 (75%, 13 knees) did not require further surgical intervention at the last follow-up.

**Conclusions:**

A single intervention of BoNT-A injections to the VL muscle combined with physiotherapy is beneficial for the treatment of patients with persistent PFPS.

**Level III evidence:**

Retrospective cohort study.

## Introduction

Patellofemoral pain syndrome (PFPS) is a common condition that may cause severe retropatellar or peripatellar pain, making it the leading cause of anterior knee pain [1]. PFPS can be provoked during extensor mechanism activation during activities such as running, climbing stairs, squatting, and cycling. PFPS is reportedly more common in women, adolescents, and athletes, and it is one of the most common running-related injuries [2]. Although it accounts for 12% of the knee-related problems seen in general orthopedic practices [3] and up to 17% of the referrals to sports medicine specialty practices [2], information on PFPS prevalence and incidence in the general population is lacking [4].

The diagnosis of PFPS is based on patient history and physical examination, and it is determined by the presentation of peripatellar or retropatellar pain that is exacerbated with loading the patellofemoral joint (PFJ) while the knee is flexed. Additional PFPS symptoms can include crepitus, tenderness on patellar facet palpation, knee effusion, knee instability, buckling, and pain while sitting that is exacerbated when standing from a sitting position [4]. Correct diagnosis and treatment of PFPS may help preserve the affected individual’s activity level, and decrease the prevalence of future patellofemoral degenerative changes [5]. PFPS has traditionally been considered a self-limiting condition, but current evidence now suggests that it may have a longer chronic course. Stathopulu et al. followed patients with PFPS during childhood and observed that 91% of them had chronic knee pain 22 years later [6].

PFPS is mainly treated non-surgically, with surgery usually being reserved for severe cases that failed to respond to non-surgical treatment [7]. While one Cochrane review found physiotherapy aimed at quadriceps strengthening to be an effective therapeutic approach [8], other modalities such as patellar taping [9], foot orthoses [10], and anti-inflammatory medications [7] were not found to be beneficial for long-term symptom relief. In contrast, other investigators have found that 40% of the patients treated by physiotherapy reported that their symptoms had persisted 1 year following treatment [11].

One of the principal mechanisms suggested for PFPS is a muscular imbalance between the vastus medialis oblique (VMO) and the vastus lateralis (VL) muscles. Previous studies have shown the benefit of botulinum type A (BoNT-A) injections not only on spastic disease [12, 14], but also to weaken selected muscle antagonist or agonist. BoNT-A is one of seven strains of the neurotoxin produced by *Clostridium botulinum*. Following intramuscular injection, BoNT-A produces a functional “denervation” via inhibition of acetylcholine release at the neuromuscular junction [15]. In animal models, muscle function recovery occurs over a 3 month period, due initially to formation of new terminal nerve sprouts and eventually to recovery of the parent terminal [15]. Therefore in theory, with the application of BoNT-A, the over-activated muscle tone of VL will be inhibited and the muscle imbalance between VMO and VL can then be altered, thus effectively correcting improper patellar tracking.

The objective of this study was to determine whether injecting botulinum type A (BoNT-A) to the VL muscle in combination with physiotherapy targeting the VMO muscle would alter the muscle balance and be an effective treatment for patients with anterior knee pain who had exhausted conservative treatment. We hypothesize that a single injection of BoNT-A to the VL muscle combined with physiotherapy will improve the patients with persistent PFPS.

## Materials and methods

### Study design

A retrospective review was performed on 26 consecutive patients (31 knees) aged 22–84 (average age 49.9) years who were treated with BoNT-A injections to the VL muscle followed by physiotherapy at a single tertiary medical center between 2008 and 2015 and met the inclusion criteria. Data on patient demographics and the presence of coexisting osteoarthritis were recorded. The patients were diagnosed with PFPS based on history, physical examination, and assessment of advanced diagnostic imaging [17], all performed by the same experienced orthopedic surgeon. The physical examination included an active patella instability, which involved holding the knee in 15° of flexion while the patient contracted the quadriceps muscle. A patellar lateral subluxation of more than 5 mm was regarded as a positive test result [19]. All patients had bilateral anteroposterior standing and lateral radiographs as well as a dynamic computerized tomographic (CT) scan consisting of four protocols: full extension with and without quadriceps contraction, and a 15° flexed knee scan with and without quadriceps contraction [17].

BoNT-A injections were offered as an alternative to surgery to the patients who met all of the following inclusion criteria: (1) history of pain characteristic of PFPS, (2) radiological evidence of patellar subluxation, and (3) exhaustion of non-surgical treatment for more than a year. Radiologic assessment of patellar subluxation was made by measuring a congruence angle > 16° (formed by a line bisecting the sulcus angle and a line drawn from the median ridge of the patella to the deepest point of the intercondylar sulcus) and > 2 mm distance between the margins of the medial pole of the patella and the medial trochlear facet or the median ridge of the patella with respect to the apex of the trochlear sulcus [17, 18]. Patients who presented with concurrent knee pathology outside of the patellofemoral joint (PFJ) and those who did not meet the minimum of 6 months of follow-up were excluded from this analysis. The present study was conducted upon receiving approval from the institutional review board (IRB).

### Procedures

**Fig. 1:**
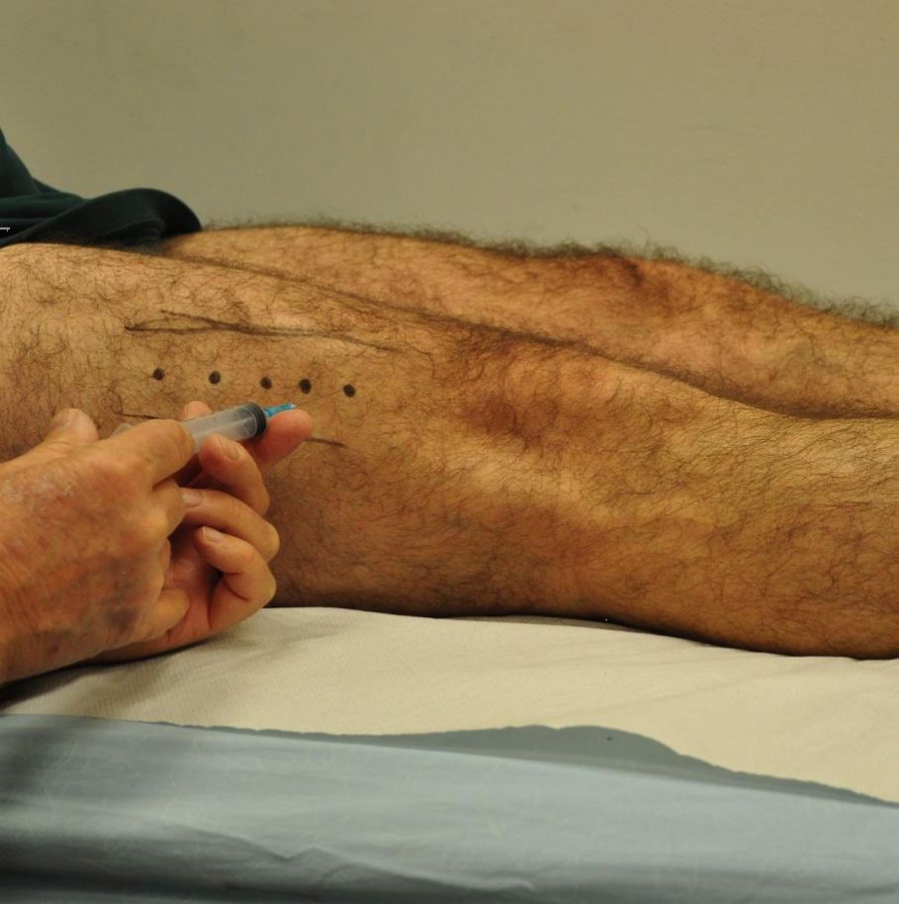
BoNT-A injection site

The subjects were placed in a supine position. The patients received 500 units of BoNT-A (Dysport®; Ipsen Biopharmaceuticals) divided into five injections that were administered into the distal part of the VL muscle at 3 cm intervals apart beginning 3–5 cm above the patella, on an oblique angle just lateral to the midline [19]. This was performed as an outpatient office procedure without image guidance (Fig. 1). Following the injections, the patients were started on a VMO muscle strengthening protocol utilizing open and closed kinematic chain exercises. A clinical follow-up evaluation was performed every 3 months.

### Outcome measures

Patients were asked to complete functional and pain assessment questionnaires. The Kujala [20] and Lysholm Knee Scoring Scales [21] were used for functional assessment. The numerical rating scale (NRS) [22] and the SF-6D [26] questionnaires were used to evaluate pain and health-related quality of life. The primary outcome was defined as the delta change in the patient reported outcome measures (PROMs) prior to and following the intervention. The pre- and post-intervention analysis included comparisons of four subgroups (age, gender, presence of PFJ osteoarthritis, and compliance to post-intervention physiotherapy). The effectiveness of intervention in each subgroup was assessed by measuring the delta improvement in the scores of each questionnaire. The secondary outcome was avoidance of a pre-planned surgical intervention for PFPS based on the improvement achieved by the combined BoNT-A treatment and completed physiotherapy protocol.

### Statistical analysis

All statistical analyses were performed using SPSS v24 (IBM Corporation, Armonk, New York). Paired-sample *t*-tests (normally distributed data) or related-sample Wilcoxon signed rank tests (skewed data) were used to compare changes between pre- and post-operation questionnaire scoring. Comparisons between sub-comparison groups (age, gender, coexisting osteoarthritis, and physiotherapy) were analyzed using independent sample *t*-tests (normally distributed data) or independent sample Mann–Whitney *U* tests (skewed data). Pearson (normally distributed data) or Spearman correlations (skewed data) were used to analyze the correlations between pre-operation questionnaire scores and changes in the questionnaire scores following the operation. Lastly, stepwise linear regression models were conducted to predict changes in questionnaire scores following the procedure.

## Results

### Patient characteristics

A total of 26 patients (31 knees) were included in the study, 10 females and 21 males. The average age was 50.1 years (range: 22–84 years). The mean follow-up was 58.8 months (range: 11–104 months). Sixteen knees (52%) were right-sided and 15 (48%) were left-sided (Table 1). Coexisting osteoarthritis of the PFJ was confirmed by CT in 18 (58%) knees prior to the initiation of treatment.

**Table 1:** Baseline characteristics of study participants

Characteristics	Participants(*n* = 26, knees = 31)
Age*, years	50.1 (22–84)
Follow up time*, months	58.8 (11–104)
Male: female, *n*, (%)	10: 21 (32%: 68%)
Injected leg (R: L), *n*	(16: 15)
Osteoarthritis: yes: no (%)	18: 13 (58%: 42%)

^*^Values are expressed as mean (range)

### Outcome measurements

#### Primary outcome

The cohort showed statistically significant improvement in all patient reported outcomes (Table 2). The mean Lysholm score improved by 27.0 ± 17.6 points, the Kujala score improved by 23.8 ± 17.0 points, the SF-6D score improved by 0.2 ± 0.2 points, and the NRS score was reduced by 4.4 ± 3.0 points. (*P* < 0.001 for all comparisons). These score differences are all clinically relevant as the established minimal clinically important difference (MCID) value pertaining to patellofemoral disorders for the Lysholm Knee Scoring Scale, Kujala, and SF-6D have been previously proposed as 10.1, 11.9, and 0.05, respectively 24–26.

**Table 2:** Outcomes of the intervention

Questionnaire	Pre (mean ± SD) Post (mean ± SD)	Δ (mean ± SD)	*P*−value
Lysholm	56.2 ± 15.0	27.0 ± 17.6	< 0.001
	83.2 ± 16.5		
Kujala	58.9 ± 12.6	23.8 ± 17.0	< 0.001
	82.7 ± 15.2		
NRS	7.6 ± 2.0	−4.4 ± 3.0	< 0.001
	3.2 ± 3.0		
SF-6D	0.6 ± 0.1	0.2 ± 0.2	< 0.001
	0.8 ± 0.2		

*NRS *numerical rating scale

##### Subgroup analysis

When the patients were divided into two subgroups based on age (< 60 and ≥ 60 years), there were statistically significant differences between all measured pre- and post-intervention scores evaluated (*P* < 0.05). Age was the sole variable to independently influence questionnaire score improvement in a linear regression model. Specifically, there was an inverse linear relation between patients’ age and the Lysholm, Kujala, and SF-6D scores, but not the NRS score. The mean pre- and post-intervention Kujala and Lysholm scores in females were slightly lower compared with males. However, both males and females showed statistically significant differences between their pre-intervention compared with post-intervention scores for all of the measured variables (*P* < 0.05). Eighteen of the study participants had coexisting osteoarthritis compared with 13 who did not. Pre-intervention compared with post-intervention scores statistically differed for both these groups when considering all measured variables (*P* < 0.05).

Twenty-five of the 31 study knees (81%) completed the physical therapy protocol aimed to strengthen the VMO muscle after the BoNT-A injections, and the remaining 6 (19%) did not. Both subgroups showed statistically significant differences in Lysholm, Kujala, and NRS pre-intervention scores compared with their post-intervention scores (*P* < 0.05). However, with regards to SF-6D scores, only the subgroup that completed the physiotherapy protocol had statistically significant differences between their pre- and post-intervention scores. All of these findings are summarized in Table 3.

**Table 3:** Subgroup analysis—the intervention effectiveness in each group

Questionnaire	Age group		Gender		Osteoarthritis		Physiotherapy	
	< 60 years	≥ 60 years	Male	Female	Yes	No	Yes	No
	(*n* = 18)	(*n* = 13)	(*n* = 10)	(*n* = 21)	(*n* = 18)	(*n* = 13)	(*n* = 25)	(*n* = 6)
Lysholm								
Pre	56.4 ± 16.9	61.5 ± 10.4	58.7 ± 17.6	55.0 ± 14.0	57.9 ± 12.9	53.8 ± 17.8	54.9 ± 14.5	61.6 ± 17.3
Post	82.6 ± 18.8	84.0 ± 13.4	89.8 ± 11.5	80.1 ± 17.9	80.7 ± 19.1	86.7 ± 12.1	82.5 ± 17.0	86.0 ± 15.8
P^**^	< 0.001	0.004	0.002	< 0.001	< 0.001	< 0.001	< 0.001	0.021
Kujala								
Pre	55.3 ± 12.9	63.9 ± 10.9	62.0 ± 16.0	57.5 ± 10.8	61.5 ± 12.0	55.3 ± 13.1	57.6 ± 12.1	64.6 ± 14.5
Post	82.3 ± 17.0	83.2 ± 13.0	90.3 ± 11.2	79.1 ± 15.8	81.6 ± 16.5	84.2 ± 13.7	81.4 ± 15.0	88.2 ± 16.5
P^**^	< 0.001	< 0.001	0.002	< 0.001	< 0.001	< 0.001	< 0.001	0.032
NRS								
Pre	7.7 ± 1.6	7.4 ± 2.5	6.9 ± 1.7	7.9 ± 2.1	7.9 ± 2.1	7.2 ± 1.9	7.7 ± 2.1	7.2 ± 1.5
Post	3.2 ± 3.1	3.2 ± 2.9	2.8 ± 3.0	3.4 ± 3.0	3.6 ± 3.1	2.7 ± 2.8	3.3 ± 3.1	3.0 ± 2.8
P^**^	< 0.001	0.001	0.002	< 0.001	< 0.001	< 0.001	< 0.001	0.042
SF-6								
Pre	0.6 ± 0.1	0.7 ± 0.1	0.6 ± 0.1	0.6 ± 0.1	0.6 ± 0.1	0.6 ± 0.1	0.6 ± 0.1	0.7 ± 0.2
Post	0.8 ± 0.2	0.8 ± 0.1	0.9 ± 0.1	0.7 ± 0.2	0.7 ± 0.2	0.8 ± 0.1	0.8 ± 0.2	0.8 ± 0.1
P^**^	< 0.001	0.019	0.02	< 0.001	0.005	0.001	< 0.001	0.171

All data are presented as mean ± SD

P**- *p*-value of the comparison inside each subgroup. Refers to the Paired samples *t*-test (normal distribution) or related samples Wilcoxon signed rank test (skewed distribution)

Although not statistically significant, there were improvements in all questionnaire scores irrespective of age, gender, presence of osteoarthritis with PFJ, and compliance to physiotherapy among all subgroups that underwent BoNT-A treatment (Table 4). The scores of the younger subgroup (< 60 years) showed greater improvement compared with the older subgroup (≥ 60 years) with regard to all measured variables. Female patients reported lower improvements in all measured scores compared with males. The presence or absence of osteoarthritis did not statistically affect the delta improvement for all questionnaire scores. Those who were compliant with the physiotherapy protocol showed a slightly higher improvement in Lysholm and Kujala scores compared with patients who were not, but these findings were not statistically different. Additionally, correlation analyses were performed to check the influence of the pre-intervention scores on measured improvement. The lower the Kujala, Lysholm, NRS, and SF-6D pre-intervention scores were, the greater the post-intervention improvement.

**Table 4:** Subgroup analysis—improvement ∆ comparison between the subgroups

	Subgroups	Lysholm	Kujala	NRS	SF-6
Age group	< 60 years	30.5 ± 19.0	27.2 ± 18.3	−4.5 ± 3.0	0.2 ± 0.2
	≥ 60 years	22.5 ± 15.5	18.9 ± 14.4	−4.2 ± 3.2	0.1 ± 0.1
	P^*^	0.17	0.183	0.76	0.071
Gender	Male	31.0 ± 22.9	28.7 ± 21.3	−4.1 ± 3.1	0.2 ± 0.2
	Female	25.3 ± 15.1	21.4 ± 14.6	−4.5 ± 3.1	0.1 ± 0.1
	P^*^	0.574	0.268	0.753	0.297
Osteoarthritis	Yes	22.8 ± 14.6	19.8 ± 14.5	−4.3 ± 3.0	0.1 ± 0.1
	No	33.2 ± 20.6	29.3 ± 19.3	−4.5 ± 3.2	0.2 ± 0.1
	P^*^	0.097	0.128	0.871	0.153
Physiotherapy	Yes	27.8 ± 18.1	23.8 ± 16.8	−4.4 ± 2.9	0.1 ± 0.1
	No	24.4 ± 18.0	23.5 ± 19.5	−4.2 ± 3.8	0.2 ± 0.1
	P^*^	0.827	0.975	0.869	0.854

∆ Data represent the differences between the post- and pre-intervention scores. Values are expressed as mean ± SD

P* the *p*-value of the subgroup comparison within each group. Refers to the independent sample *t*-test (normal distribution) or independent sample Mann–Whitney *U* test (skewed distribution)

#### Secondary outcome

Sixteen patients (18 knees) were awaiting surgical intervention prior to the course of BoNT-A injections upon exhausting nonsurgical treatments over a period of 1 year. At the last follow-up, 12 patients (75%, 13 knees) did not elect to undergo surgery owing to satisfactory pain relief and improved function. During all follow-up visits, no patient presented with complications or side effects related to the BoNT-A treatment.

## Discussion

The results of the present study elucidate that BoNT-A injections may be an effective and safe treatment for patients with persistent PFPS at midterm follow-up, regardless of the patient’s age, gender, coexisting osteoarthritis, or compliance with physiotherapy following treatment. PFPS is a common knee pathology that negatively impacts an individual’s QOL. An increased pressure on the PFJ, which can be related to a quadriceps muscle imbalance, likely causes it. Specifically, the etiology lies between the VMO muscle, which is the active medial stabilizer of the patella to the VL muscle, the iliotibial band, and the retinaculum, all of which apply forces in the lateral direction [27].

It has been suggested that the imbalance of the quadriceps muscle can be caused by a reduction of the VMO muscle force or by compromised temporal control of the VMO and VL muscle activity [28, 29]. Cowan et al. reported that PFPS patients demonstrated a delayed onset of electromyographic activity of the VMO muscle relative to the VL muscle [30]. In patients with a PFPS, the VL was activated earlier than the VMO when patients climbed downstairs and upstairs; however, in the control group that imbalance did not exist [30]. Although an objective parameter such as electromyographic activity was not evaluated in the present study, the clinical improvement experienced by patients may be adequate to justify BoNT-A as an effective option in treating PFPS. These findings have been supported by several other studies [29, 31, 32]. One previous study suggests that patients with patellofemoral problems exhibited atrophy of the VMO [33]. Furthermore, Van Tiggelen et al. in their prospective study determined that an activation delay of the VMO muscle was a risk factor to the development of PFPS in healthy soldiers [34]. Cloudon et al. demonstrated that the peak knee extension movement of PFPS patients was impaired compared with controls and that it reached normal values after directed treatment [35]. In addition, Besier et al. showed that the knee extension moments were reduced in PFPS patients during running compared with healthy controls [36]. Therefore, it is reasonable to assume that aiming to correct the VMO:VL.

One experimental way of changing the VMO:VL ratio is by injecting BoNT-A into the VL muscle combined with a tailored exercise program, thus preventing patellar maltracking through the achievement of normal muscle balance [19, 37–40]. BoNT-A is a neurotoxin that weakens the muscle by blocking the pre-synaptic acetylcholine release, allowing weakness relative to the given dose of a muscle to be achieved for 12 weeks to 6 months [41–43]. The BoNT-A action is time-limited, and the muscle function is renewed by regeneration of new nerve sprouts within one month [44]. Previous studies have established the utility of intramuscular injection of BoNT-A to address focal muscle overactivity to manage neurological conditions [45]; however, its use for treatment of musculoskeletal conditions is not as well reported in the literature [38]. In the setting of PFPS, BoNT-A injections have the potential to address the proposed imbalance between a relatively overactive VL muscle and its less active synergists, including the VMO muscle. This provides a unique opportunity for aimed muscle re-training and rebuilding of more normal quadriceps muscle activation patterns and joint function. We believe that this is more viably accomplished with explicit and centered muscle re-training fortified by expert healthcare providers, rather than just from ordinary every day movements.

To date, data on BoNT-A treatment for PFPS is relatively scarce [19, 37, 39, 40, 42, 43]. We designed this study to expand current knowledge by exploring treatment outcomes in affected patients. Singer et al. compared 14 patients treated with electromyography-guided BoNT-A injections with 10 patients who received a placebo, both followed by a physiotherapy protocol [46]. The authors reported that the treatment was beneficial in terms of improved pain and function [46]. A follow-up study of the same group showed that patient-reported improvement lasted for an average of 34 months [40]. However, it should be mentioned that both of those studies were industry-funded by Ipsen Biopharmaceuticals, the manufacturer of Dysport. Chen et al. reported on 12 patients with bilateral PFPS that showed improvement in pain, stiffness, and function of the knee after being injected with BoNT-A compared with the contralateral non-injected side at 3 months follow-up [39]. Additionally, Stephen et al. aimed to investigate the effect of an ultrasound-guided BoNT-A injection into the tensor fasciae latae (TFL) in patients with lateral patellofemoral overload syndrome (LPOS) who failed conventional treatment [47]. They concluded that an injection of BoNT-A into the TFL, combined with physical therapy resulted in a significant improvement of symptoms in patients with LPOS, which was maintained at 5-year follow-up [47].

The above-cited studies describe the use of various guiding methods to determine the injection site, which may make these treatment options more expensive and less suitable for an office procedure [37]. In our study, we used anatomic landmarks, making it more suitable for an office procedure and potentially increasing its availability. Unlike previous studies that excluded older patients (> 60 years) and patients with PFJ osteoarthritis [46], we included both of these more challenging patient populations and found that they too benefited from the BoNT-A injection and physical therapy protocol. However, the treatment was most successful in the younger age group and in patients who presented with a worse clinical status. Notably, PFPS is more common in the younger, more active population. This supports the rationale of using BoNT-A injections as a salvage therapy for patients who had exhausted the available nonsurgical treatments and who planned to undergo surgery. This was further validated in the present study as many patients in our cohort canceled their planned surgical interventions as a result of successful BoNT-A treatment. Potential adverse effect of the treatment was hypothesized to be an initial reduction in quadriceps femoris muscle strength due to the reduced contribution of the distal VL muscle. This was of concern since it has been demonstrated that significant quadriceps femoris muscle weakness may already exist in the affected limb of individuals with PFJ dysfunction [48].

There are several limitations to this study. First, due to its retrospective nature, the study was exposed to memory bias of the participants when responding to the questionnaires. The subgroup analyses may be subject to low statistical power due to the limited number of participants. Body mass index and other pre-existing comorbidities in the form of American Society of Anesthesiologists’ (ASA) classification and Charlson Comorbidity Index (CCI) were not evaluated. Additionally, a matched non-treatment cohort was not available, which would have allowed for a more in-depth analysis of the effectiveness of BoNT-A injections. Fluoroscopic or ultrasound guided injection was not utilized in the study to improve potential accuracy. A greater number of females were examined compared with male patients; PFPS is thought to more commonly affect female patients, so this may be representative [49]. Lastly, this study was performed in a single medical clinic, thus limiting its external validity. Despite these limitations, the findings of our study are promising and can be used as a platform for future investigations on this topic.

## Conclusions

Treatment of PFPS by utilizing BoNT-A injections provides a cost and time effective alternative to not only ongoing conservative management but also surgical intervention for patients experiencing refractory anterior knee pain. It significantly improved functional outcomes and reduced pain in patients who had exhausted conservative treatment methodologies. However, larger prospective studies are needed to evaluate the effectiveness of the herein described intervention and characterize the patients who will benefit most from it.

## Data Availability

The datasets generated during and/or analyzed during the current study are available from the corresponding author on reasonable request.

## Abbreviations

PFPS: Patellofemoral pain syndrome
VMO: Vastus medialis oblique muscle
VL: Vastus lateralis muscle
QOL: Quality of life
BoNT-A: Botulinum toxin A
PFJ: Patellofemoral joint
NRS: Numerical rating scale
