# Correction to: Botulinum toxin injections as a salvage therapy is beneficial for management of patellofemoral pain syndrome

**DOI:** 10.1186/s43019-022-00131-9

**Published:** 2022-02-01

**Authors:** Yuval Kesary, Vivek Singh, Tal Frenkel-Rutenberg, Arie Greenberg, Shmuel Dekel, Ran Schwarzkopf, Nimrod Snir

**Affiliations:** 1 Sackler Faculty of Medicine, Tel Aviv University, P. O. Box 39040, 6997801 Tel Aviv, Israel; 2 Department of Orthopedic Surgery, NYU Langone Health, NYU Langone Orthopedic Hospital, New York, NY USA; 3 Orthopedic Department, Rabin Medical Center, Beilinson Hospital, Petah Tikva, Israel; 4 Department of Orthopedic Surgery, Kaplan Medical Center, Rehovot, Israel; 5 Division of Adult Reconstruction, Department of Orthopedics, Tel Aviv Sourasky Medical Center, Tel Aviv, Israel

## Correction to: Knee Surg & Relat Res (2021) 33:39 https://doi.org/10.1186/s43019-021-00121-3

After publication of this article, the authors noticed an error in the Discussion and Conclusion section. The incorrect and correct information is shown below. The original article [1] has been updated. The authors apologize for any inconvenience caused.

**Table:** 

Incorrect	Correct
One experimental way of changing the VMO:VL ratio is by injecting BoNT-A into the VL muscle, combined with a tailored exercise program, thus preventing patellar maltracking through the achievement of normal muscle balance [19,37–40]. BoNT-A is a neurotoxin that weakens the muscle by blocking the presynaptic acetylcholine release, allowing dose-related weakness of a muscle to be achieved for 12 weeks to 6 months [41–43]. The BoNT-A action is time limited, and the muscle function is renewed by regeneration of new nerve sprouts within 1 month [44]. Intramuscular injection of BoNT-A to address focal muscle overactivity in the management of neurological conditions is well established [45]; however, treatment of musculoskeletal conditions is less commonly reported [38]. In the setting of PFPS, BoNT-A treatment may address the proposed imbalance between a relatively overactive VL muscle and its less active synergists, including the VMO muscle. This may provide an opportunity for focused muscle re-education and restoration of more normal quadriceps muscle activation patterns and joint function. We postulate that this will be more effectively achieved with specific, focused muscle re-education and strengthening overseen by a trained health professional, rather than just from normal daily activity.	One experimental way of changing the VMO:VL ratio is by injecting BoNT-A into the VL muscle combined with a tailored exercise program, thus preventing patellar maltracking through the achievement of normal muscle balance [19,37–40]. BoNT-A is a neurotoxin that weakens the muscle by blocking the pre-synaptic acetylcholine release, allowing weakness relative to the given dose of a muscle to be achieved for 12 weeks to 6 months [41–43]. The BoNT-A action is time-limited, and the muscle function is renewed by regeneration of new nerve sprouts within one month [44]. Previous studies have established the utility of intramuscular injection of BoNT-A to address focal muscle overactivity to manage neurological conditions [45]; however, its use for treatment of musculoskeletal conditions is not as well reported in the literature [38]. In the setting of PFPS, BoNT-A injections have the potential to address the proposed imbalance between a relatively overactive VL muscle and its less active synergists, including the VMO muscle. This provides a unique opportunity for aimed muscle re-training and rebuilding of more normal quadriceps muscle activation patterns and joint function. We believe that this is more viably accomplished with explicit and centered muscle re-training fortified by expert healthcare providers, rather than just from ordinary every day movements.
Treatment of PFPS by utilizing BoNT-A injections confers a cost- and time effective alternative to both ongoing conservative management and/or surgery for individuals with refractory anterior knee pain. It significantly improved functional outcomes and reduced pain in patients who had exhausted conservative treatment methodologies. However, larger prospective studies are needed to evaluate the effectiveness of the herein described intervention and characterize the patients who will benefit most from it.	Treatment of PFPS by utilizing BoNT-A injections provides a cost and time effective alternative to not only ongoing conservative management but also surgical intervention for patients experiencing refractory anterior knee pain. It significantly improved functional outcomes and reduced pain in patients who had exhausted conservative treatment methodologies. However, larger prospective studies are needed to evaluate the effectiveness of the herein described intervention and characterize the patients who will benefit most from it.
